# Plant-made poliovirus vaccines – Safe alternatives for global vaccination

**DOI:** 10.3389/fpls.2022.1046346

**Published:** 2022-10-20

**Authors:** Omayra C. Bolaños-Martínez, Richard Strasser

**Affiliations:** Department of Applied Genetics and Cell Biology, Institute of Plant Biotechnology and Cell Biology, University of Natural Resources and Life Sciences, Vienna, Austria

**Keywords:** expression system, molecular farming, oral vaccine, plant biotechnology, poliomyelitis

## Abstract

Human polioviruses are highly infectious viruses that are spread mainly through the fecal-oral route. Infection of the central nervous system frequently results in irreversible paralysis, a disease called poliomyelitis. Children under five years are mainly affected if they have not acquired immunity through natural infection or *via* vaccination. Current polio vaccines comprise the injectable inactivated polio vaccine (IPV, also called the Salk vaccine) and the live-attenuated oral polio vaccine (OPV, also called the Sabin vaccine). The main limitations of the IPV are the reduced protection at the intestinal mucosa, the site of virus replication, and the high costs for manufacturing due to use of live viruses. While the OPV is more effective and stimulates mucosal immunity, it is manufactured using live-attenuated strains that can revert into pathogenic viruses resulting in major safety concerns and vaccine-derived outbreaks. During the last fifteen years, plant-based poliovirus vaccines have been explored by several groups as a safe and low-cost alternative, and promising results in protection against challenges with viruses and induction of neutralizing antibodies have been obtained. However, low yields and a high frequency in dose administration highlight the need for improvements in polioviral antigen production. In this review, we provide insights into recent efforts to develop plant-made poliovirus candidates, with an emphasis on strategies to optimize the production of viral antigens.

## Introduction

Poliomyelitis (polio) is a viral disease which is caused by polioviruses that are transmitted by the fecal-oral route and predominantly affects children under five years. The severity ranges from asymptomatic occurrence to meningitis and acute flaccid paralysis. Polio has the peculiarity to seriously affect the central nervous system (CNS) and damage the motor neurons located in the anterior horn of spinal nerve roots. This harm leads to muscular dysfunction or even death when vital body functions such as deglutition or respiration are compromised (Sabin, 1956). Post-polio syndrome (PPS) is a non-contagious and slowly progressive appearance of a variety of symptoms that occur many years or decades after virus infection and involves symptoms like muscular weakness, limb paresis with muscle atrophy, paresthesia, joints pain, fatigue, physical and mental activity deterioration. The cause of PPS remains poorly understood, it may be related to the slow degeneration of individual nerve terminals in the motor units (Pastuszak et al., 2017).

Polio has a huge impact on developing countries with poor sanitation and weak public health systems. Due to worldwide vaccination efforts that began in the late ‘80s with the creation of the Global Polio Eradication Initiative (GPEI), polio has been considered almost completely eradicated. To date, polio remains endemic in two countries: Afghanistan and Pakistan (Greene et al., 2019). However, recent cases of paralytic poliomyelitis in the US, the UK and Israel highlight that poliovirus is still a worldwide threat that needs attention in all countries (Hill et al., 2022). These new cases in countries deemed polio-free were reported in undervaccinated communities which emphasizes the need to improve vaccination coverage for global polio eradication.

## Poliovirus and capsid proteins

The causative agent of the disease is poliovirus, which is a member of the *Enterovirus* genus belonging to the *Picornaviridae* family. There are three wildtype poliovirus serotypes (WPV) and all of them are highly contagious. The WPV serotypes 2 and 3 were declared as eradicated in the year 2015 and 2019, respectively. Thus, the WPV serotype 1 has become the only wild poliovirus that remains in circulation^1^ .

**Figure 1:**
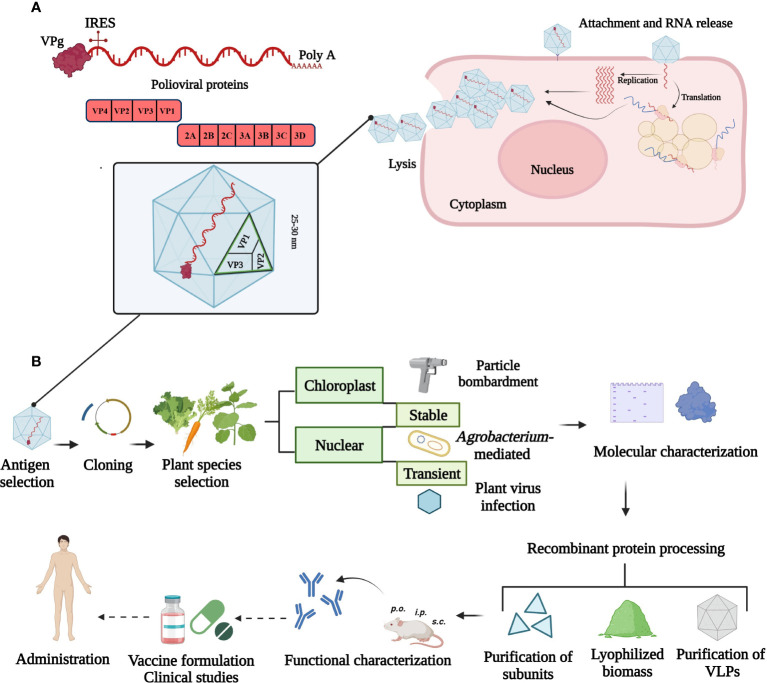
Overview of poliovirus cell infection and strategies to produce a plant-based vaccine. **(A)** Illustration of the viral genome and replication cycle in the cytoplasm. The small viral protein, VPg is attached at the 5´- and a poly-A tail at 3´-end of the ~ 7500 bp RNA. The 5´-untranslated region contains the internal ribosome entry site (IRES) for ribosome assembly. The single open reading frame encodes structural (VP1, VP2, VP3 and VP4) and non-structural (2ABC, 3ABCD) proteins. **(B)** Plant biotechnology approaches for poliovirus vaccine production. Current strategies comprise chloroplast and nuclear genome transformation using particle bombardment, *Agrobacterium*-mediated or plant virus-based expression. Unaccomplished steps towards a plant-made poliovirus vaccine are marked with dotted arrows. This figure was created with BioRender.com.

The full-length poliovirus genome has approximately 7500 bp and is composed of a single-stranded, positive-sense and non-segmented RNA. The major ORF is flanked by two untranslated regions containing a variety of secondary structures. The small viral protein VPg is attached covalently to the 5´-end and a long chain of adenine residues is attached to the 3´-end of the RNA. Both modifications are involved in replication and translation processes in association with membranous complexes (Yogo and Wimmer, 1972; Lee et al., 1977). Viral RNA released into the cytoplasm serves as messenger RNA (mRNA) from which VPg is cleaved off. The ribosomes assemble on the internal ribosome entry site (IRES) and the mRNA is subsequently translated to a single polyprotein that is proteolytically processed into mature viral proteins. The replication process and virions assembly occur in the cytoplasm of infected cells where virally encoded and host cell proteins are required (Mueller et al., 2005; Payne, 2017) (**Figure 1A**).

Poliovirus has an icosahedral morphology resulting from integration of 60 copies of each of the four structural (capsid) proteins named VP1, VP2, VP3 and VP4 (Jiang et al., 2014). The VP4 protein resides inside of the virus particle and has a myristic acid moiety attached at the N-terminus which has an important role for virus assembly (Paul et al., 1987). The VP1, VP2, and VP3 proteins are exposed outside of the viral structure and contain the main antigenic sites, which are defined as linear or non-linear structures where the neutralizing antibodies can bind and prevent infection (Hogle et al., 1985; Fry and Stuart, 2010; Shaw et al., 2018). The RNA genome is encapsidated into these non-enveloped icosahedral virions in a process that involves membrane rearrangements and hijacking of cellular proteins involved in membrane transport in the secretory pathway (Belov et al., 2007). Unlike many enveloped viruses like coronaviruses, the biosynthesis of the polioviral proteins and assembly of the virions typically does not involve contact with the luminal biosynthesis machinery of the secretory pathway. Hence, the exposed polioviral proteins are not N-glycosylated and N-glycosylation likely does not play any role in poliovirus protein folding, secretion or function.

## Host immune response and current vaccines to prevent poliomyelitis

After ingestion, the virus replicates in the alimentary tract mucosa and is transported by macrophages and/or infected dendritic cells to deep cervical lymph nodes and mesenteric lymph nodes where the virus replicates (Wahid et al., 2005). The viruses are released into the lymph and transported through the afferent lymphatic vessels into the bloodstream, causing a primary viremia and subsequent viral spread to other tissues. In approximately, 1% of individuals who either have not been vaccinated or have not acquired natural immunity, the circulating viruses can invade the CNS causing an irreversible paralysis (Blondel et al., 2005). To date, two vaccines are administered: 1) the inactivated polio vaccine (IPV) and 2) the live-attenuated oral polio vaccine (OPV). The production of these vaccines is based on conventional procedures involving handling large amounts of infectious viruses which are subsequently inactivated or attenuated. These vaccines have become the main tool for global eradication efforts of the disease. Both vaccines result in systemic immunity with the production of IgG and monomeric IgA antibodies that circulate in the body and protect from paralytic polio. Additionally, OPV induces intestinal mucosal immunity that prevents infections in the gut and protects the gastrointestinal and urogenital tracts. This immunity is associated with the production of secretory IgA antibodies (Donlan and Petri, 2020). While IPV-vaccinated individuals are protected from the disease, they may still shed poliovirus which results in virus transmission and thus contributes to the spread of viruses in communities. Limitations of the OPV, on the other hand, are related to the generation of vaccine-associated paralytic polio. Due to genetic instability, the live-attenuated virus can regain virulence leading to the production of new pathogenic strains called circulating vaccine-derived poliovirus (cVDPV) and increased outbreaks in recent years (Ming et al., 2020; Alleman et al., 2021). As a response, in 2020 the World Health Organization (WHO) approved the use of the novel OPV monovalent type 2 (nOPV2) vaccine, a genetically more stable vaccine produced form the Sabin type 2 strain. While this vaccine is safer, the current nOPV2 supply is limited^2^. Hence, the current scenario raises the urgent need for new strategies towards polio eradication where advanced virus-free vaccines are designed that provide effective protection and prevent the transmission of poliovirus and the reemergence of the disease.

## Biotechnology strategies for the production of plant-based vaccines

During the last three decades, plant biotechnology has experienced a huge progress in producing economically valuable and biologically active heterologous proteins. A myriad of biopharmaceuticals, including monoclonal antibodies, diagnostic reagents and viral antigens used as vaccine prototypes for animals and humans have been produced in plants. In this respect, a diversity of plant species such as tobacco, tomato, soybean, potato, rice, maize and carrot have been explored as vaccine biofactories and oral delivery vehicles (Shanmugaraj et al., 2020). Viruses from which small protein antigens or complete proteins have been expressed include human papillomavirus (HPV), human immunodeficiency virus (HIV), viruses from the genus *Flavivirus* (Dengue virus, Zika virus, West Nile virus), hepatitis B virus (HBV), porcine reproductive and respiratory syndrome virus (PRRSV), food and mouth disease virus (FMDV), Newcastle disease virus (NDV), rabies virus (RABV), influenza virus, coronaviruses-like swine transmissible gastroenteritis virus (TGEV) and SARS-CoV-2 (Mason et al., 1992; Berinstein et al., 2005; Ashraf et al., 2005; Zhou et al., 2008; Rybicki, 2014; Uribe-Campero et al., 2015; Gottschamel et al., 2016; Massa et al., 2019; Bolaños-Martínez and Rosales-Mendoza, 2020; Ruocco and Strasser, 2022). In comparison to conventional expression systems such as bacteria or mammalian cells, plants offer unique features. For viral proteins, folding is highly essential to maintain the antigenic conformation of the epitopes and retain their capacity to induce a desired immune response. Bacterial expression systems are limited in processing of proteins and lack mammalian N- and O-glycosylation capacity. As a consequence, appropriate protein folding often cannot be achieved (Sahdev et al., 2008). Plants, on the other hand, combine the characteristics of higher eukaryotic cells and are capable of proper post-translational modifications and efficient protein folding. The production is highly scalable and safe since plant cells are not infected by human pathogens like viruses and are free of undesirable biological contaminants such as endotoxins or prions. Furthermore, plant-based production offers lower manufacturing costs compared to mammalian cells and the possibility to generate freeze-dried formulations that are stable without cooling (Fischer et al., 2004; Kumar et al., 2018).

Plant-based vaccines can be produced by delivering a transgene into the plastid (stably) or nuclear genome (stably or transiently) or by using RNA-based viral expression systems (**Figure 1B**). Edible plants like carrot or lettuce lack toxic compounds and require minimal processing for the formulation of oral vaccines (e.g. delivery *via* tablets). These formulations are especially important to elicit a mucosal immune response at sites where polioviruses replicate. In addition, natural encapsulation and adjuvant-intrinsic effects attributed to components of plant cells can further enhance the effect of expressed viral antigens, for example, by protecting the antigen or enabling a controlled release (Rosales-Mendoza and Tello-Olea, 2015). Transient expression is attractive for emerging viruses and can offer high protein yields in a short time period (Ruocco and Strasser, 2022). Advantages of chloroplast expression include reduced variation from positional effects, lack of gene silencing, polycistronic expression and the possibility of open field cultivation of engineered crops because of the maternal inheritance of the chloroplast genome. High protein expression levels can be achieved due to the high number of DNA copies per chloroplast and high number of organelles per leaf cell (Daniell, 2006; Zhang et al., 2017). A limitation of chloroplasts is their bacterial origin leading to limited post-translational modifications and problems with protein folding. Transgenic and transplastomic plants as well as transient expression approaches have been successfully used to produce poliovirus antigens and virus-like particles (VLPs) in plants.

## Plant-made poliovirus vaccine candidates

The approach of using plants to produce a recombinant vaccine is in accordance with the objectives proposed by the Global Polio Eradication Initiative (GPEI), covering the optimization of oral vaccines and developing an affordable inactivated vaccine. Several research groups have explored plant-based production for the development of polio vaccine candidates (**Table 1**). In 2006, Fujiyama et al., 2006 fused a fragment of 15 amino acids derived from the VP3 and VP1 capsid proteins (Sabin type 1 strain) to the tobacco mosaic virus (TMV) coat protein. The fusion protein was expressed in a transient form in tobacco plants obtaining a yield of up to 0.2 mg/g of fresh leaves. Mice were immunized intraperitoneally once or twice with 200 µg of purified recombinant TMV particles emulsified in monophosphoryl lipid A and trehalose dimycolate as adjuvants. The intraperitoneal immunization with the chimeric TMV particles resulted in induction of specific antibodies (Fujiyama et al., 2006).

**Table 1:** Compilation of plant-made vaccine prototypes against polioviruses.

Plant	Antigen used	Expression strategy	Immunization scheme	Immunogenic and protective potential	References
*Nicotiana tabacum*	First 11 amino acids from the C-terminus of VP3 and 4 amino acids from the N-terminus of VP1 (Sabin 1)	Transient	Groups of 3 C57BL mice immunized i.p. with 200 µg of recombinant virus particles emulsified in monophosphoryl lipid A and trehalose dimycolate	Specific-peptide antibodies induced in sera	Fujiyama et al., 2006
*Nicotiana tabacum*	VP1 and CTB-VP1 (Sabin 1)	Stable (Chloroplast)	Groups of 10 CD-1 mice were primed with IPV, then an oral booster was given once a week for 8 consecutive weeks with 20 mg of plant tissue adjuvanted with saponine, squalene or both	Titers of specific IgG and IgA antibodies increased, neutralizing antibody titers and seropositivity between 70-90% against the three Sabin strains obtained	Chan et al., 2016
*Nicotiana tabacum*	VP1 and CTB-VP1 (Sabin 1)	Stable (Chloroplast)	Groups of 10 CD-1 mice were primed with IPV followed by 12 oral boosters with 1 or 25 μg of VP1 once a week for 8 consecutive weeks, then once a month for 3 months and one after 6 months. The doses were adjuvanted with saponine, squalene or both	Elevated titers of IgG1 and IgA antibodies induced and maintained from 29 to 400 days	Xiao and Daniell, 2017
*Nicotiana benthamiana*	Polyprotein P1 (PV3 SktSC8 mutant)	Transient	Groups of 8 TgPVR mice received 1 or 2 i.p. injections of purified VLPs corresponding to the equivalent of half a human dose, then a second dose was administered on day 14	VLPs with the native D antigenic conformation generated. Neutralizing antibodies elicited and protection against wild PV3 challenge developed	Marsian et al., 2017
Lettuce	VP1 and CTB-VP1 (Sabin 1)	Stable (Chloroplast)	Groups of 10 CD-1 mice were primed with IPV followed by 6 oral boosters with 20 mg of plant tissue performed every 2 weeks for 2 months and 2 every 3 months. The doses were adjuvanted with saponine, squalene, plus the antimicrobial compounds PG-1 or LL37	VLPs with 22.3 nm in size generated. Specific IgG and IgA antibodies were enhanced with VP1 oral boosters	Daniell et al., 2019
*Nicotiana tabacum*	VP1, VP2, VP3 and VP4 (Sabin 1)	Stable (Nuclear)	Groups of 5 BALB/c mice received 4 s.c. immunizations once a week followed by 4 oral boosters every 2 weeks. The s.c. and oral doses contained 0.8 μg of VP1, 1.40 μg of VP2, 0.43 μg of VP3 or 0.60 μg of VP4 without adjuvants.	Specific IgG and secretory IgA antibodies elicited against all VP proteins	Bolaños-Martínez et al., 2020
Carrot	VP1 and VP2 (Sabin 1)	Stable (Nuclear)	Groups of 4 BALB/c mice were immunized following two different schedules: 1) 4 s.c. immunizations once a week, or 2) 4 s.c. immunizations once a week followed by 5 oral boosters once a week for 4 consecutive weeks plus one final 150 days after. The s.c. and oral doses contained 0.11 μg of VP1 or 1.38 μg of VP2 without adjuvants.	Specific IgG and secretory IgA long-lasting antibodies were detected in the groups immunized with VP1 or VP2 proteins following the s.c. plus oral route	Bolaños-Martínez et al., 2022

i.p., intraperitoneally; s.c., subcutaneous.

Chloroplast-based expression was used to produce the VP1 capsid protein fused to the cholera toxin B subunit in tobacco plants (expression level: 2.6 mg/g dry weight) (Chan et al., 2016). The immunogenicity was tested in mice in a scheme comprising the subcutaneous administration of the IPV followed by oral boosters with freeze-dried plant material adjuvanted with squalene, saponine or both. The titers of specific IgG and IgA antibodies significantly increased in sera from mice fed with the plant tissue compared with lower titers when no boosters were administered. Additionally, neutralizing activity and seropositivity (70-90%) against the three Sabin serotypes was observed with two doses of IPV followed by plant-made VP1 protein oral boosters. In a long-term study for this vaccine booster, 1 or 25 μg of adjuvanted VP1 protein was orally given to mice first primed with IPV (Xiao and Daniell, 2017). High levels of IgG1 and IgA antibodies were induced and the immune response was sustained for 400 days with protection against the three poliovirus serotypes during the time period.

The generation of poliovirus VLPs containing the capsid proteins was first reported by Marsian et al., 2017. The polyprotein P1 from the Sabin type 3 mutant SktSC8 was expressed transiently in *Nicotiana benthamiana* together with a polyprotein processing proteinase. Stable VLPs were generated (yield up to 60 µg/g) that retained the native, immunogenic D antigenic conformation. Transgenic mice expressing the poliovirus receptor were intraperitoneally immunized with purified plant-produced VLPs carrying poliovirus antigens. Importantly, the immunized mice showed protection after challenge with a wild poliovirus 3 strain and structural analysis of the VLPs demonstrated a morphology resembling native polioviruses (Marsian et al., 2017). In a later study, Daniell et al., 2019 developed transplastomic lettuce lines suitable for oral immunization. VP1 was assembled as VLP of approximately 22.3 nm in size. Specific IgG1 and IgA antibodies as well as neutralization activity was observed in mice first primed with IPV and thereafter with three oral boosters with 20 mg of lyophilized lettuce material adjuvanted with squalene, saponine or both plus antimicrobial peptides to enhance the immune modulation.

Bolaños-Martínez et al., 2020 expressed the VP1, VP2, VP3 and VP4 proteins to enable the poliovirus Sabin type 1 capsid formation. The four capsid proteins were expressed in leaves of transgenic tobacco and for each protein the antigenicity was shown. Expression levels ranging from 0.3 µg/g to 16.85 µg/g of fresh leaves were obtained. The immunological potential of the plant-made capsid proteins was further determined by immunizing mice in a scheme comprising subcutaneous and oral boosters with lyophilized plant material containing the four capsid proteins (Bolaños-Martínez et al., 2020). Humoral systemic and mucosal antibody responses were generated with VP1, VP3 and VP4 proteins, while the VP2 protein was less efficient in stimulating specific IgG antibody production.

In order to investigate the capacity of a different edible plant to yield poliovirus VPs and advance to the development of an oral vaccine candidate, the VP1 and VP2 Sabin 1 proteins were expressed in carrot cells (Bolaños-Martínez et al., 2022). The obtained yields ranged from 1.17 to 3.57 µg/g fresh weight for the VP1 protein and 4.24 to 13.86 µg/g for the VP2 protein. Upon immunization, mucosal and systemic responses were obtained when a schedule combining parenteral priming and oral boosting without external adjuvants was applied to BALB/c mice. Interestingly, the presence of specific IgG and secretory IgA antibodies was detected even 212 days after the start of the immunization schedule indicating a long-lasting protection.

## Future directions

To achieve global poliomyelitis eradication, innovative vaccines are needed since the actual vaccines are produced with infectious or attenuated poliovirus strains that raise safety concerns. Endemic and vaccine-derived polio cases are mainly observed in developing or low-income countries which urges the need to develop affordable and accessible vaccines. Transient expression in plants provides a fast and flexible approach to produce vaccines in case of newly emerging viral pathogens as shown by the current COVID-19 pandemic or in cases where a vaccine has to be adapted quickly to a mutating virus. For genetically more stable viruses, transgenic expression could be cheaper and provide a constant supply for vaccination. Developing an edible vaccine that can be used for priming or booster oral immunization and non-infectious VLPs appear as the most promising strategies for a plant-produced polio vaccine. Although huge progress has been made with evidence of humoral response induction that led to neutralization of polioviruses when evaluated in challenge assays (Marsian et al., 2017), none of the studies has progressed towards clinical phase. One reason is that the expression levels and yields of plant-produced poliovirus proteins are still quite low. Transient co-expression of human chaperones like calreticulin has been successfully applied to increase the yields of glycosylated viral proteins (Margolin et al., 2020; Rosenberg et al., 2022). The cytosolic chaperone HSP90 plays an important role in folding and maturation of poliovirus capsid proteins (Geller et al., 2007). Additional expression of human chaperones like HSP90 could therefore be tested to increase the overall expression levels. For chloroplast-based production, co-expression of a bacterial chaperone like CesT could be considered (MacDonald et al., 2017). Moreover, seed-based expression systems could be investigated to increase protein stability and yields. In addition to their excellent storage properties, seeds are natural reservoirs of nutrients and ideal sources for oral vaccine formulation (Rosales-Mendoza et al., 2017; Schwestka et al., 2020).

The promising data from pre-clinical studies using plant-based production together with the demand for high vaccination coverage and the limitations with currently approved polio vaccines show the huge potential for plant-based polio vaccines. In February 2022, a plant-based COVID-19 vaccine consisting of coronavirus VLPs manufactured by Medicago and an adjuvant was approved by Health Canada (Hager et al., 2022). This is a milestone as it shows for the first time the safety and efficacy of a plant-produced vaccine against a human infectious virus. We are convinced that the COVID-19 vaccine approval will pave the way for the next generation of plant-made vaccines including hopefully one that is functional and cost effective against polio.

## Author contributions

All authors listed have made a substantial, direct, and intellectual contribution to the work and approved it for publication.

## Funding

This work was supported by the Austrian Science Fund (FWF) Project P31920-B32.

## Conflict of interest

The authors declare that the research was conducted in the absence of any commercial or financial relationships that could be construed as a potential conflict of interest.

## Publisher’s note

All claims expressed in this article are solely those of the authors and do not necessarily represent those of their affiliated organizations, or those of the publisher, the editors and the reviewers. Any product that may be evaluated in this article, or claim that may be made by its manufacturer, is not guaranteed or endorsed by the publisher.

## Footnotes

1
https://polioeradication.org/

2
https://polioeradication.org/
